# A Review of Acute Myocardial Injury in Coronavirus Disease 2019

**DOI:** 10.7759/cureus.8426

**Published:** 2020-06-03

**Authors:** Anupama B K, Debanik Chaudhuri

**Affiliations:** 1 Internal Medicine, State University of New York (SUNY) Upstate Medical University, Syracuse, USA; 2 Interventional Cardiology, State University of New York (SUNY) Upstate Medical University, Syracuse, USA

**Keywords:** sars-cov-2, covid-19, acute myocardial injury, cardiac troponin, myocarditis

## Abstract

In December 2019, an outbreak of pneumonia caused by a novel coronavirus, severe acute respiratory syndrome coronavirus 2 (SARS-CoV-2), occurred in Wuhan, Hubei province, China, and it has spread rapidly across the world, causing the coronavirus disease 2019 (COVID-19) pandemic. Although SARS-CoV-2 infection predominantly results in pulmonary issues, accumulating evidence suggests the increased frequency of a variety of cardiovascular complications in patients with COVID-19. Acute cardiac injury, defined as elevated cardiac troponin levels, is the most reported cardiac abnormality in COVID-19 and strongly associated with mortality. In this article, we summarize the currently available data on the association of SARS-CoV-2 and COVID-19 with acute myocardial injury.

## Introduction and background

Severe acute respiratory syndrome coronavirus 2 and Coronavirus disease 2019

Coronaviruses are enveloped non-segmented positive-sense ribonucleic acid (RNA) viruses belonging to the family Coronaviridae, which are broadly distributed in humans and other mammals, and cause respiratory, enteric, hepatic, and neurologic disease [1]. Six coronavirus species were previously known to cause human disease [1]. Four viruses-229E, OC43, NL63, and HKU1-typically cause mild respiratory illness in immunocompetent individuals; whereas, the other two betacoronaviruses-severe acute respiratory syndrome coronavirus (SARS-CoV) and Middle East respiratory syndrome coronavirus (MERS-CoV)-have been linked to fatal illnesses in the past two decades [1,2]. SARS-CoV was the causal agent of the severe acute respiratory syndrome outbreaks in 2002 and 2003 in Guangdong province, China. MERS-CoV was the pathogen responsible for severe respiratory disease outbreaks in 2012 in the Middle East and has been responsible for more than 10,000 cumulative cases in the past two decades; mortality rates of 10% for SARS-CoV and 37% for MERS-CoV have been reported [1-3].

In December 2019, the first pneumonia cases of unknown origin were identified in Wuhan, the capital city of Hubei province, China. These cases were epidemiologically linked to a local Huanan wholesale seafood market [1,2]. A previously unknown betacoronavirus was discovered through unbiased sequencing in samples from patients with pneumonia. Human airway epithelial cells were used to isolate a novel enveloped RNA betacoronavirus, named 2019-nCoV, and later renamed severe acute respiratory syndrome coronavirus 2 (SARS-CoV-2) formed a clade within the subgenus sarbecovirus, orthocoronavirinae subfamily [1]. Phylogenetic analysis showed that SARS-CoV-2 has 89% genome sequence identity to a bat SARS-like coronavirus, 80% identity to SARS and 50% identity to MERS coronavirus, thus making SARS-CoV-2 the seventh member of the coronavirus family that infects humans, as well as the third coronavirus with bat origins [4].

Since its initial identification, the disease caused by SARS-CoV-2, coronavirus disease 2019 (COVID-19) has spread to more than 187 countries worldwide over the past few months [5]. Given the rapid spread of this virus, with consequences on an international scale, COVID-19 was declared a pandemic by the World Health Organization on March 11, 2020 [6]. As of May 10, 2020, more than four million COVID-19 cases were reported globally (including more than 1.3 million cases in the United States), which are associated with more than 281,000 deaths to date [5]. Although SARS-CoV-2 appears to have a lower fatality rate than either SARS-CoV or MERS-CoV, COVID-19 has resulted in many more deaths than both of these prior outbreaks combined, partly because of its greater infectivity (estimated reproductive number (R0) of between 2 and 3) and higher attack rate, thus leading to more infected patients [6]. Evidence of person to person transmission has been observed, primarily through close contact and respiratory droplets. The virus can be detected one to two days before symptom onset in upper respiratory samples, and the median incubation period has been estimated to be 5.1 days (95% confidence interval (CI), 4.5-5.8 days) [7]. Although most symptomatic patients with COVID-19 present with fever, dry cough and shortness of breath, and show pneumonia on imaging findings, approximately of ten percent of patients have a worsening of the disease, thus requiring intensive care and possible complications such as acute respiratory distress syndrome (ARDS), viremia, acute cardiac injury, disseminated intravascular coagulation (DIC), multi-organ failure and subsequent death in critically ill patients [8].

Definition of acute myocardial injury

Myocardial injury is defined as an elevation in cardiac biomarkers, cardiac troponin I (TnI) or troponin T (TnT) above the 99th percentile of the upper reference limit, and is considered acute if there is a rise and/or fall in cardiac troponin concentrations exceeding the biological and/or analytical variation; myocardial injury may be secondary to ischemic or nonischemic processes [9,10]. Traditionally, elevated troponin concentrations have been considered equivalent to myocardial infarction. However, with improvements in troponin assays, elevated levels without overt symptoms or signs of myocardial ischemia are now more common; hence, the fourth universal definition of myocardial infarction considers myocardial injury to be a separate, unique entity [11]. Based on current evidence, a myocardial injury without overt ischemia represents approximately 60% of cases of abnormal troponin elevation [9]. The differential diagnosis is broad in such cases. It can be recognized in the variety of cardiac such as acute heart failure, pulmonary embolism, myocarditis, cardiac surgery or procedures, cardiac arrhythmias, hypertension, stress-induced cardiomyopathy, or several non-cardiac conditions such as acute renal failure, sepsis, anemia, hypoxia, critical illness, drug-induced, rhabdomyolysis among others [9,10]. Association of viral infections with myocardial injury has been well recognized, and the most common associations involve adenoviruses and enteroviruses such as coxsackie viruses [12]. According to data from previous influenza virus and coronavirus epidemics, these viral infections have also been shown to cause myocardial injury, because cases of myocarditis have been described to be caused by both influenza and coronaviruses [13,14].

Although SARS-CoV-2 infection predominantly causes pulmonary complications, such as pneumonia and ARDS, the disease has also been associated with a variety of cardiovascular complications, including acute myocardial injury, myocarditis, arrhythmia, heart failure, and venous thromboembolism [6]. Moreover, studies have shown that patients with COVID-19 and preexisting cardiovascular disease (CVD) have an increased risk of acquiring the illness and subsequently having severe disease and death [6,7]. In a clinical bulletin issued by the American College of Cardiology (ACC), the overall case-fatality rate was 2.3%, but the mortality reached 10.5% in patients with underlying CVD [15]. Among various cardiac complications, acute cardiac injury with elevated cardiac biomarkers has been described in early studies from China on hospitalized patients with COVID-19. We discuss the prevalence, mechanism, clinical characteristics, management, and prognostic implications of SARS-COV-2-induced myocardial injury, according to current published research.

## Review

Cardiovascular complications with SARS-COV and MERS

SARS-CoV and MERS may also result in cardiovascular complications, although most of the data are anecdotal, in the absence of systematic studies. Although SARS-CoV infection is associated with some cardiovascular manifestations, there are no clear reported cases of myocarditis [16-19]. However, in the 2012 Saudi Arabia outbreak, MERS-CoV was reported to cause acute myocarditis and heart failure (Table 1) [14].

**Table 1:** Cardiovascular complications with SARS and MERS SARS: severe acute respiratory syndrome; MERS: Middle East respiratory syndrome

Outbreaks	First author and cohort size	Cardiovascular manifestation	Outcomes
SARS	Yu et al. [16] (n=121)	Tachycardia (72%), hypotension (50%), bradycardia (15%), cardiomegaly (11%) and paroxysmal atrial fibrillation in only one patient	Transient
	Pan et al. [17] (n=15)	Cardiac arrest	Death
	Li et al. [18] (n=46)	Subclinical diastolic dysfunction without systolic impairment	Reversible on recovery
	Peiris et al. [19] (n=75)	Acute myocardial infarction	Cause of death in two of five fatal cases
MERS	Alhogbani [14] (n=1)	Acute myocarditis and acute onset heart failure	Recovered

Prevalence of myocardial injury in COVID-19

Acute cardiac injury has been reported to be highly prevalent in hospitalized patients with COVID-19 (Table 2).

**Table 2:** Prevalence of myocardial injury in COVID-19 and patient outcomes COVID-19: coronavirus disease 2019; ICU: Intensive care unit

First author and cohort size	Type of study	Acute cardiac injury	Outcome
Huang et al. [1] (n=41)	Single center, retrospective case series	Present in five (12%) patients	Four patients required ICU
Wang et al. [20] (n=138)	Single center, retrospective case series	Present in ten patients (7.2%) overall, 22% of whom required ICU care	Eight patients required ICU
Zhou et al. [21] (n=191)	Multicenter, retrospective cohort	Present in 33 patients (17%)	Of 33 patients, only one survived
Liu et al. [22] (n=291)	Single center, retrospective	Present in 15 patients (5.2%)	Of 15 patients, 11 required ICU, and one died
Xu et al. [23] (n=53)	Single center, retrospective	Present in six patients (11.3%)	All required ICU, and two died
Shi et al.[24] (n=416)	Single center, cohort	Present in 82 patients (19.7%)	Higher mortality in patients with cardiac injury (42 of 82, 51.2%) vs patients without cardiac injury (15 of 334, 4.5%)
Guo et al. [25] (n=187)	Single center, retrospective, case series	Present in 52 patients (27.8%)	In-hospital mortality 59.6% (31 of 52) in patients with elevated troponin T levels, compared with 8.9% (12 of 135) in patients with normal troponin T levels

The earliest single-center retrospective study conducted in China, involving 41 hospitalized patients with COVID-19, detected acute cardiac injury in five (12%) patients. In the study, a diagnosis of acute cardiac injury was made if serum levels of cardiac biomarkers (troponin I) were above the 99th percentile upper reference limit, or new abnormalities were detected in electrocardiography (ECG) and echocardiography [1]. Since then, numerous retrospective studies reported an increased prevalence of myocardial injury in hospitalized patients with COVID-19, with a frequency between 5% and 28% [1,20-25]. The prevalence of cardiac injury increased with the severity of the disease (Table 3) [26-31].

**Table 3:** List of studies demonstrating increased prevalence of myocardial injury in patients with severe COVID-19 COVID-19: coronavirus disease 2019; TnI: troponin I

First author and cohort size, type of study	Patient classification	Findings
He et al. [26] (n=54) single center retrospective study	All patients with severe or critical COVID-19	Twenty-four (44.4%) patients had myocardial injury complications. In hospital mortality was significantly higher in patients with than without myocardial injury (14(60.9%) vs 8(25.8%), p=0.013)
Hiu et al. [27] (n=41), single center retrospective study	A total of two, 32, four and three patients were clinically diagnosed with light, mild, severe, and critical COVID-19, respectively	Four patients had elevated troponin, including one severe case and three critical cases; no elevation was observed in light and mild cases (p<0.01). The peak value of cardiac TnI in critical cases was 40-fold above the normal value. Computed tomographic imaging of epicardial adipose tissue (EAT) was used to demonstrate the cardiac inflammation; low EAT density was observed in severe and critical cases.
Zhou et al. [28] (n=34), single center retrospective study	Eight patients met the criteria for very severe COVID-19, and 26 met the criteria for severe COVID-19	Significantly increased cardiac biomarkers including cardiac TnI in the very severe group compared with the severe group. Cardiac TnI was elevated in all eight patients in the very severe group and only one of 26 patients in the severe group (p<0.001)
Chen et al. [29] (n=150), single center retrospective study	Among patients with COVID-19, 24 met the criteria for the critically ill group, and 126 met the criteria for the non-critically ill group	Significantly higher levels of cardiac TnI were observed in critically ill patients than in non-critically ill patients (p<0.05).
Lippie et al. [30] (n=341), metanalysis of four studies	One hundred twenty-three (36%) patients with severe COVID-19 disease	Significantly higher cardiac TnI levels in patients with severe disease than without severe disease (standardized mean difference, 25.6 ng/L; 95% CI, 6.8–44.5 ng/L, p<0.001).
Han et al. [31] (n=274), single center retrospective study	Patients with COVID 19 were divided into three groups: mild (198 cases), severe (60 cases) and critical (15 cases)	Significantly greater positive rate of ultra-TnI in severe cases and critical case than mild cases (p<0.05)

However, data regarding troponin elevation in asymptomatic and mildly symptomatic, non-hospitalized patients are not available.

Mechanism of cardiac injury in COVID-19

The mechanism of cardiac injury among patients with COVID-19 remains uncertain. The following potential mechanisms have been suggested.

1. SARS-CoV-2 Induced Direct Myocardial Injury

One potential mechanism is direct myocardial involvement mediated via angiotensin-converting enzyme 2 (ACE2) [8,32]. The betacoronaviruses can infect human hosts through ACE2, a membrane-bound protein expressed in many human cells, including vascular endothelia, renal tissue, cardiovascular tissue, and small intestinal epithelia. ACE2 negatively regulates the renin-angiotensin system by inactivating angiotensin II, and it is likely to protect against acute lung failure. A murine model has demonstrated that pulmonary infection with SARS-CoV also precipitates ACE2‐dependent myocardial infarction. Among humans, during the Toronto SARS outbreak, SARS-CoV viral RNA was detected in 35% of autopsied hearts, thus raising the possibility of direct damage of cardiomyocytes by the virus [32]. Phylogenetic analysis has demonstrated that SARS-CoV-2 has a very similar receptor binding domain/motif to that of the SARS coronavirus, thus suggesting that SARS-CoV-2 may use ACE2 as a receptor to enter human cells and causes direct damage to the lung epithelia, thereby leading to pneumonia and ARDS. Because ACE2 is also highly expressed in the heart, COVID-19 induced cardiac injury might potentially be mediated by ACE2, although whether SARS-CoV-2 binding alters ACE2 expression or causes dysregulation of the renin-angiotensin-aldosterone system (RAAS) pathway remains unclear [6,7,32,33]. Hence, one potential explanation for the higher likelihood of acquiring infection, and the increased risk of severe disease and adverse outcomes in patients with COVID-19 with pre-existing CVD, maybe the elevated secretion of ACE2 in these patients, thus making them more susceptible to direct viral damage to cardiac myocytes [33]; but, this has not yet been demonstrated in pathology studies. In a case report describing autopsy results for a 50-year-old man who died of COVID-19 associated with ARDS, histologic examination of biopsied cardiac tissue showed rare interstitial mononuclear inflammatory infiltrates without substantial myocardial damage. However, the troponin level was not reported in the study [34]. 

2. SARS-CoV-2 Induced Overwhelming Inflammatory Response and Cytokine Storm

Another suggested mechanism of COVID-19 related cardiac involvement is an overwhelming immune-inflammatory response and cytokine storm [6,7,33]. The hallmark of cytokine storm syndrome is an uncontrolled and dysfunctional immune response involving the continuous activation and proliferation of lymphocytes and macrophages [24]. Huang et al. have found that patients with COVID-19 admitted to the intensive care unit have higher plasma levels of cytokines, including interleukins (IL-2, IL-7, IL-10, granulocyte-colony stimulating factor and IgG-induced protein 10), monocyte chemoattractant protein-1, macrophage inflammatory protein 1-alpha, and tumor necrosis factor α [1]. From a previous study of SARS-CoV infection and cardiovascular complications, subclinical diastolic left ventricular impairment appears to be common during acute SARS infection and to be reversible after recovery, thus suggesting that left ventricular dysfunction in the acute phase might be associated with cytokine storm syndrome [18]. Several studies have shown that patients with COVID-19 with myocardial injury show evidence of severe systemic inflammation, including elevated leukocyte and neutrophil counts, interleukin levels, C-reactive protein, procalcitonin, globulin, and biomarkers of myocardial injury and stress, such as creatine kinase and myoglobin and N-terminal pro-B-type natriuretic peptide (NT-proBNP), thus linking myocardial injury to the severity of inflammation and subsequent ventricular dysfunction [24,25,35]. Similarly, in a case report of SARS-CoV 2 induced fulminant myocarditis, the described patient had markedly elevated interleukin 6, thus suggesting that the presence of cytokine storms might have caused increased vascular wall permeability and myocardial edema, thus resulting in the observed thickening of the interventricular septum. Because the myocarditis was transient, and recovery was enhanced with the use of continuous renal replacement therapy (CRRT), myocardial injury can be considered to be associated with an excessive immune response mediated by viral infection [36].

3. SARS-CoV-2 Induced Severe Hypoxia and Systemic Disturbances

Severe hypoxia due to acute lung injury and several systemic complications caused by the virus can result in oxidative stress and hence secondary myocardial injury due to increased myocardial oxygen demand [7,33]. For example in one study, there was a characteristic pattern of rise in cardiac troponin I over time. The troponin level was normal during admission, but subsequently increased in 37.5% of patients especially in those who died. In those patients, the troponin level significantly increased in the week preceding the death coinciding with severe systemic disorders including multiple organ failure, severe electrolyte disturbances, management of ventilation or extracorporeal membrane oxygenation (ECMO), severe metabolic acidosis and coagulation dysfunction all of which can potentially lead to secondary myocardial injury due to increased oxidative stress on the heart [37].

Preexisting cardiovascular disease and acute myocardial injury in SARS-CoV-2

Studies have demonstrated that patients with underlying CVD and other comorbid conditions are predisposed to myocardial injury during COVID-19; the prevalence of hypertension among patients with cardiac injury has been reported to be as high as 45%-65%, and that of coronary artery disease (CAD) has been reported to be approximately 20%-30% (Table 4) [22,24,25].

**Table 4:** Prevalence of preexisting cardiovascular disease in patients with COVID-19 COVID-19: coronavirus disease 2019; COPD: chronic obstructive pulmonary disease; N/A: not available

Comorbidities	Studies, size of cohort and p values					
	Shi et al. [24], (n=416), p<0.05		Guo et al. [25], (n=187), p<0.05		Liu et al. [22] (n=291), p<0.05	
	With cardiac injury (n=82)	Without cardiac injury (n=334)	With cardiac injury (n=52)	Without cardiac injury (n=135)	With cardiac injury (n=15)	Without cardiac injury (n=276)
Hypertension	59.8%	23.4%	63.5%	20.7%	46.6%	17%
Coronary artery disease	29.3%	6.0%	32.7%	3.0%	20%	3.3%
Diabetes	24.4%	12.0%	30.8%	8.9%	20%	6.9%
Chronic heart failure/cardiomyopathy	14.6%	1.5%	15.4%	0	6.7%	0
Cerebrovascular disease	15.9%	2.7%	N/A	N/A	N/A	N/A
COPD	7.3%	1.8%	7.7%	0	N/A	N/A

In addition to the above mechanisms, in patients with underlying CVD, the acute systemic inflammatory responses seen in COVID-19 can exacerbate inflammatory activity within coronary atherosclerotic plaques, thus making the plaques prone to rupture. Inflammation also causes endothelial dysfunction and increases the procoagulant activity of the blood, thereby contributing to the formation of occlusive thrombi over ruptured coronary plaques and predisposing patients to ischemic myocardial injury [38].

COVID-19 and myocarditis

There are several clinically diagnosed myocarditis cases in patients with COVID-19 with supporting imaging (Table 5) [36,39-41]. 

**Table 5:** Case reports on SARS-CoV-2 induced myocarditis SARS-CoV-2: severe acute respiratory syndrome coronavirus 2; TnI: troponin I; TnT: troponinT; hs-TnT: hish sensitivity troponin T; CK: creatine kinase; NTBNP: n-terminal brain natriuretic peptide; NT-proBNP: N-terminal pro-B-type natriuretic peptide; ARDS: acute respiratory distress syndrome; MODS: multiple organ dysfunction syndrome; IL-6: Interleukin-6; LVEF: left ventricular ejection fraction; CT: computed tomography; CAD: coronary artery disease; MRI: magnetic resonance imaging; CRRT: continuous renal replacement therapy; ECMO: extracorporeal membrane oxygenation; VA-ECMO: Veno arterial ECMO; IABP: Intra-aortic balloon pump; N/A, not available; RT PCR: real‐time reverse transcriptase‐polymerase chain reaction assay

Case reports Author name, publication date	Age (years)	History of preexisting cardiac condition	Chief complaint	Diagnosis of SARS-CoV-2 (real- time RT PCR assay)	Markers of myocardial injury	Associated complications	Investigation			Treatment	Outcome
							Electrocardiography	Transthoracic echocardiogram	Others		
Zeng et al. [36], Infection, April 10, 2020	53	None	Fever, cough, shortness of breath and chest tightness	Sputum sample positive for SARS-CoV-2	Elevated TnI, NTBNP	Severe pneumonia, ARDS, MODS, remarkably high IL- 6 (272.40 pg/mL)	Sinus tachycardia	Diffuse myocardial dyskinesia, enlarged left ventricle, LVEF (32%)	N/A	Methylprednisolone, Immunoglobulin, lopinavir-ritonavir, Interferon α-1b, antibiotics and ventilatory support CRRT and ECMO on day 11	LVEF recovery to 68%, and left ventricle wall thickness restored to the normal range. Decreased TnI, NTBNP and IL-6 (7.63 pg/ml)
Hu et al. [39], European Heart Journal, March 16, 2020	37	None	Chest pain, dyspnea and diarrhea for 3 days	Sputum sample positive for SARS-CoV-2	Elevated TnT, CK-MB, NTBNP	Pneumonia and cardiogenic shock	ST-segment elevation in lead III, AVF	Cardiomegaly, LVEF 27%.	CT coronary angiography; no coronary stenosis.	Methylprednisolone, Immunoglobin, antibiotics and medical treatment for heart failure and cardiogenic shock	Recovery of cardiac chamber dimensions to normal range. Normalization of myocardial injury markers after 3 weeks
Inciardi et al. [40], JAMA Cardiology, March 27, 2020	53	None	Severe fatigue	Nasopharyngeal swab positive for SARS-CoV-2	Elevated hs- TnT, CK-MB, NT-proBNP	Cardiogenic shock but no evidence of pneumonia	Diffuse ST elevation	Diffuse hypokinesis, LVEF 40%	Coronary angiography; no evidence of obstructive CAD Cardiac MRI- acute myopericarditis	Methylprednisolone, lopinavir/ritonavir, hydroxychloroquine, aspirin and medical treatment for heart failure	Improvement in LVEF to 44% on day six, along with a significant decrease in LV wall thickness and slight decrease in pericardial effusion; clinical and hemodynamic improvement.
Tavazzi et al. [41], European Journal of Heart Failure, April 10, 2020	69	N/A	Persistent cough, progressive dyspnea and fatigue for 4 days	Nasopharyngeal swab positive for SARS-CoV-2	Elevated hs-TnI	Cardiogenic shock, respiratory failure, severe metabolic acidosis	N/A	Severe diffuse hypokinesia, dilated left ventricle, LVEF 25%, estimated cardiac index 1.4 L/kg/min	Coronary angiography unremarkable Endomyocardial biopsy; low grade myocardial inflammation and viral particles in the myocardium	IABP, mechanical ventilation, VA-ECMO	Recovery of left ventricular function initially, ECMO, IABP weaned on day 5, death due to septic shock from gram negative pneumonia without evidence of left ventricular dysfunction

In the study, among 84 patients with COVID 19, four (4.8%) were clinically diagnosed with SARS-CoV-2 myocarditis based on the most updated diagnostic criteria for viral myocarditis [42]. However, in a single-center retrospective study in 112 admitted patients with confirmed COVID-19, the authors explored whether SARS-CoV-2 caused the myocarditis by performing at least one echocardiographic evaluation in all patients during hospitalization. A total of 14 patients (12.5%) presented with abnormalities suggestive of possible myocarditis with an elevation of troponin. The myocarditis diagnosis was based on findings of triple elevation in cardiac troponin I (>0.12 ng/mL) plus abnormalities in echocardiography and/or ECG. The abnormalities in echocardiography were defined as a reduced left ventricular ejection fraction (LVEF) (<50%) or segmental wall motion abnormality or left ventricular wall thickening (>10 mm) and/or presence of pericardial effusion (≥5 mm); the abnormalities in ECG were defined as ST-segment elevation/ST-T changes. The echocardiography did not show typical signs of myocarditis, as stated above, except for the presence of small pericardial effusion in some patients and nonspecific ECG manifestations, most commonly tachycardia, which was not typical for myocardial injury but was more suggestive of systemic causes. Hence, the authors suggested that myocardial injury may be more likely to be a result of systemic consequences rather than direct damage by SARS-CoV-2 [37]. 

Uncertainties exist whether SARS-CoV-2 can directly cause cardiomyocyte infection as no biopsy- or autopsy-demonstrated SARS-CoV-2 localization within cardiomyocytes has been reported to date. In a study of COVID-19 through post-mortem needle core biopsy of the lung, liver, and heart in four patients who died of COVID-19 pneumonia, cardiac biopsy performed in two patients in whom cardiac troponin was elevated during hospital course showed only mild focal fibrosis and mild myocardial hypertrophy, which were probably associated with the underlying conditions, such as hypertension-associated myocardial hypertrophy and past ischemic injury; however, there was no evidence of inflammatory cellular infiltration to indicate myocarditis [43].

Tavazzi et al. have described the first case in which a biopsy demonstrated myocardial localization of viral particles suggestive of SARS-CoV-2, in a patient presenting with cardiogenic shock [41]. The clinical presentation was suggestive of fulminant myocarditis; however, the pathologic study demonstrated low-grade myocardial inflammation without evidence of myocardial necrosis. Given that the viral particles were observed in interstitial cytopathic macrophages and their surroundings, but not in cardiac myocytes, the authors could not infer viral cardiotropism and suggested that either a viremic phase or migration of infected macrophages from the lung is likely to occur in patients with COVID‐19 with non‐ischemic acute myocardial injury [41]. Further investigations along with the histological demonstration of myocarditis (inflammatory lymphomonocytic infiltrates plus myocyte necrosis not typical of ischemia [44]) and identification of the viral genome in cardiac tissue will be required in the future to clarify the direct association of COVID-19 with myocarditis.

Patterns of myocardial injury in COVID-19

Myocardial injury has been shown to present in three different patterns:

1. The most common pattern is mild troponin elevation (typically < 99th percentile of the upper reference limit), with a modest rise or fall on subsequent days, as frequently observed in patients without cardiac symptoms and those who survive after hospitalization [21,37].

2. The second pattern is progressive, in which some patients have normal troponin levels or moderate troponin elevation during admission. However, the level progressively increases as the patients experience clinical deterioration with respiratory failure along with an increase in other biomarkers (e.g., interleukin-6, ferritin, and lactate dehydrogenase) and usually occurs during the second week of hospitalization. This pattern is observed among non-survivors, and death occurs around a median of 18.5 days after symptom onset. One study has demonstrated that at four days after symptom onset, the median high sensitivity cardiac TnI (hs-cTnI) levels were 8.8 pg/mL in non-survivors vs. 2.5 pg/mL in survivors. During follow-up, the median hs-cTnI among survivors did not change significantly (2.5-4.4 pg/mL), whereas it rose to 24.7 pg/mL on day 7, to 55.7 pg/mL on day 13, to 134.5 pg/mL on day 19 and to 290.6 pg/mL on day 22 in non-survivors [21,32,37].

3. The third pattern is early moderate troponin elevation (which may approach or exceed the 99th percentile upper reference limit) and, subsequently, fall over the course of illness; this pattern is usually seen in patients with clinically suspected myocarditis presenting predominantly with cardiac symptoms [36,39,40].

Clinical features of patients with COVID-19 and myocardial injury

Studies have shown that patients with cardiac injury are more likely to be older (median age 65-74 years) than those without cardiac injury [22,24,25], and most patients present with typical symptoms of COVID-19 like fever, cough, fatigue, and dyspnea. In a study of 291 COVID-19 patients, 15 of whom had evidence of cardiac injury, no patients complained of chest pain or palpitations. The common symptoms at illness onset in both groups of patients with or without cardiac injury were similar and included fever, cough, headache, fatigue, and dyspnea [22]. However, a minority of patients with cardiac injury might present with cardiac symptoms with or without respiratory symptoms. In another study, 11 of 82 (13.4%) patients with myocardial injury presented with chest pain, compared with 3 of 334 (0.9%) patients without cardiac injury (P<0.001) [24]. In a case report of myocarditis, the initial presenting symptom was chest pain and respiratory involvement, but an atypical presentation without respiratory symptoms was also described [36,37]. In terms of radiologic findings, bilateral and multiple mottling and ground-glass opacity are more prevalent in patients with than without cardiac injury [25]. Limited studies in which patients with cardiac injury underwent ECG have revealed ECG abnormalities such as T-wave depression and inversion, ST-segment depression, and Q waves [24]. Patients with clinically suspected myocarditis have been found to have ST-segment elevations [39,40].

Complications

Patients with cardiac injury and elevated troponin are more likely to be admitted to the intensive care unit and to develop complications including ARDS, malignant arrhythmias including ventricular tachycardia/ventricular fibrillation, acute coagulopathy, electrolyte disturbances, and acute kidney injury, and to receive mechanical ventilation, CRRT, extracorporeal membrane oxygenation, and vasopressor therapy, thus further suggesting that cardiac injury might be associated with the clinical outcomes of COVID-19. The use of antibiotic treatment, glucocorticoids, and intravenous immunoglobulin treatment is also significantly higher in patients with cardiac injury than without cardiac injury [24,25].

Myocardial injury and mortality in patients with COVID-19

Studies have shown that cardiac injury is associated with higher mortality in patients with COVID 19 (Tables 2,3). In a case series study in 416 patients with COVID-19, the Cox regression model, after adjustment for age, preexisting cardiovascular diseases, and other comorbid conditions and complications including ARDS, revealed a significantly higher risk of death in patients with than without cardiac injury, either during the time from symptom onset (hazard ratio [HR], 4.26 [95% CI, 1.92-9.49]) or time from admission to study endpoint (HR, 3.41 [95% CI, 1.62-7.16]) [24]. Similarly, In another case series, even though patients with COVID-19 with cardiac injury and underlying CVD had the highest mortality (69.44%), patients with underlying CVD but normal TnT levels during the disease course had more favorable prognoses than patients with elevated TnT levels but no underlying CVD (mortality, 13.3% vs. 37.5%), thus indicating relatively favorable prognosis in patients with underlying CVD but without myocardial injury [25]. These studies demonstrate that cardiac injury might be independently associated with an increased risk of mortality in patients with COVID-19.

Moreover, the mortality rate directly increases with the magnitude of the reference value of cardiac troponin. In a single-center, retrospective cohort study including 188 patients with COVID-19 in Wuhan, China, conducted to explore whether heart injury occurred during COVID-19 on admission and later increased mortality, approximately 11.2% of patients had high-sensitivity cardiac troponin I (hs-TnI) exceeding the clinical upper normal limit on admission. Patients with high levels of hs-TnI on admission had significantly higher mortality (50.0%) than patients with moderate or low levels of hs-TnI (10.0% or 9.1%). Besides, hs-TnI level on admission was significantly negatively correlated with the number of survival days (r=-0.42, p=0.005) [35]. A study by Guo et al. showed that TnT and NT-proBNP levels increased significantly during hospitalization in patients who ultimately died, whereas these dynamic changes in TnT or NT-proBNP levels were not evident in survivors [25]. Based on a few studies, initial measurement of cardiac biomarkers immediately after hospitalization for SARS-CoV-2 infection and longitudinal monitoring during the hospital stay may aid in identifying a subset of patients who might progress to a poorer clinical condition. However, further evidence from more studies is required.

Management considerations for acute myocardial injury in patients with COVID-19

A myocardial injury should be suspected in patients with COVID-19 with troponin elevation or new abnormalities in ECG and could be related to type 1 or type 2 myocardial infarction, myocarditis, stress-induced cardiomyopathy or cytokine induced-myocardial injury [45,46]. The approach to evaluating myocardial injury in patients with known or suspected COVID-19 should be based on balancing the benefits of further evaluation in changing the management strategy versus the potential risk of nosocomial spread of COVID-19 infection [45,46].

As such, elevated troponin levels should not be considered as evidence of acute myocardial infarction, which is diagnosed based on symptoms and signs of ischemia and ECG changes [9]. In a multicenter case series study form six hospitals in New York inpatients with confirmed COVID-19 who had ST-segment elevation on ECG with the elevation of cardiac enzymes, 10 out of 18 patients were classified as having noncoronary myocardial injury with no evidence of obstructive disease on coronary angiography and normal wall motion on echocardiography. Eight patients received a clinical diagnosis of myocardial infarction. The median peak troponin level was higher in patients with myocardial infarction than in those with a clinical diagnosis of noncoronary myocardial injury [47]. 

If the clinical presentation is suggestive of an acute coronary syndrome (ACS), timely evaluation is required to determine the need for urgent intervention. In patients with COVID-19 who have mild to moderate troponin elevation without symptoms and signs of acute heart failure, clinical monitoring may be performed without routine cardiac imaging until the recovery from the acute viral syndrome [46]. For patients with clinical signs and symptoms of heart failure, echocardiography may be performed if the management strategy and prognosis would be likely to change with the diagnosis. The possible diagnosis in the patients with ventricular wall motion abnormalities, elevated troponin, and no ACS include clinically suspected myocarditis or stress cardiomyopathy [45]. As there is no established therapy for clinically suspected myocarditis, routine evaluation with cardiovascular magnetic resonance or endomyocardial biopsy in those cases is not recommended [45]. As such, regardless of fulminant or non-fulminant presentation, American college of cardiology recommends against routine endomyocardial biopsy in patients with active COVID-19 with abnormal cardiac enzymes as biopsy is unlikely to change immediate clinical management whether the etiology is due to myocarditis, cytokine-induced myocardial injury or type- 2 myocardial infarction [45,46].

Unfortunately, beyond guidelines for treating patients with ischemic myocardial injury, no consensus exists regarding the routine management of patients with COVID-19 and myocardial injury. The management of myocardial injury focuses on identifying and treating the underlying cause and supportive care (including management of heart failure, therapy of arrhythmias, and avoidance of cardiotoxins) [45,46]. If the patient clinically deteriorates or develop hemodynamic instability, or has severely elevated or rapidly rising troponin, detailed evaluation, cardiology consultation and enrollment in clinical trials or experimental therapies like antiviral agents, anti-cytokines (IL-6 inhibitors such as tocilizumab), the convalescent plasma may be considered on the case-by-case basis [46]. Although the use of steroids has not been recommended in the general treatment of COVID-19, some reports have described patients recovering from fulminant myocarditis with intravenous immunoglobulin and steroids. However, more studies will be needed to determine which patient would benefit from such therapies [8]. For patients with refractory shock or ventricular arrhythmias secondary to fulminant myocarditis, early institution of mechanical support with ECMO can be considered as case reports with the successful rescue of such patients have been described [46]. In general, as the presence of CVD and myocardial injury in COVID-19 patients has been associated with more severe outcomes and increased mortality, triaging patients with COVID-19 according to the presence of underlying CVD and evidence of myocardial injury for prioritized treatment strategies may be reasonable [25].

Finally, several therapies with potential cardiovascular side effects are currently being studied, and caution must be used when administering these therapies to patients with underlying cardiovascular disease. For example, preliminary clinical research has suggested the potential efficacy of hydroxychloroquine alone and in combination with azithromycin for treatment of COVID-19, but this is associated with QTc prolongation and risk of fatal arrhythmias, thus warranting regular monitoring of QTc [48]. Controversy remains concerning the use of ACEI/ARB for COVID-19. Some preclinical studies have suggested that RAAS inhibitors may increase ACE2 (functional receptor for SARS-CoV-2) expression, raising concerns regarding safety in patients with COVID-19; however, the data remain anecdotal, and no clinical studies have evaluated the effects of RAAS inhibitors in COVID-19. As such, several leading professional societies recommend continuation of RAAS inhibitors for patients who are at risk for, being evaluated for or currently have COVID-19, and are taking these medications to treat other conditions for which these agents are known to be beneficial, such as heart failure, or ischemic heart disease [49].

Future directions

Most studies have been conducted on Chinese cohorts, and thus the generalizability of the results to other countries, including the United States, remains to be determined. Most studies have been single-centered retrospective studies with the enrollment of small numbers of patients. Larger cohorts, multicenter and prospective studies should be planned. They should include assessment of detailed heart function related data, particularly ECG, echocardiography and possibly cardiovascular magnetic resonance imaging to determine myocardial function, and the etiology and mechanism of cardiac injury. Most studies have not discussed why troponin was measured in the first place, and no data are available to support a different management strategy for patients with elevated troponin. Studies must be planned to define whether adjunctive cardioprotective therapies, such as corticosteroids, immunosuppressants, antivirals agents, interferons and/or immunomodulatory therapy (immunoglobulins), may be advisable for patients with significant elevation of cardiac injury biomarkers. Pathological examination of myocardial tissue of patients with COVID-19 with definite myocardial injury will be needed to provide pathological evidence of the etiology of myocardial injury and establish COVID-19 as a new etiological agent for myocarditis.

## Conclusions

In conclusion, COVID-19 has been associated with an increased prevalence of acute cardiac injury, even more so in patients with severe disease. The presence of preexisting cardiovascular disease increases the risk of cardiac injury in patients with COVID-19. Although the exact mechanism underlying the association of cardiac injury in patients with COVID-19 remains unclear, direct virus-induced cardiomyocyte injury leading to viral myocarditis, systemic cytokine-mediated myocardial injury and hypoxia-induced myocardial oxidative stress have been suggested. The presence of myocardial injury is independently associated with mortality and may be a predictor of progression to severe disease and adverse clinical outcomes in patients with COVID-19. Further studies are needed to elucidate the predominant etiology of myocardial injury in patients with COVID-19 and to promote targeted treatment programs to improve patient prognosis.
